# Morphologically controlled synthesis of ferric oxide nano/micro particles and their catalytic application in dry and wet media: a new approach

**DOI:** 10.1186/s13065-017-0278-0

**Published:** 2017-05-31

**Authors:** Muhammad Ramzan Saeed Ashraf Janjua, Saba Jamil, Nazish Jahan, Shanza Rauf Khan, Saima Mirza

**Affiliations:** 1 Department of Chemistry, King Fahd University of Petroleum and Minerals (KFUPM), Dhahran, 31261 Kingdom of Saudi Arabia; 2 Laboratory of Superlight Materials and Nano Chemistry, Department of Chemistry, University of Agriculture, Faisalabad, 38000 Pakistan; 3 Punjab Bio Energy Project of Punjab Government, University of Agriculture, Faisalabad, 38000 Pakistan

**Keywords:** Nanostructures, Chemical synthesis, Solvent effect, Thermo gravimetric analysis (TGA), Catalytic properties, Nitrophenol, Pollutant, Reduction

## Abstract

Morphologically controlled synthesis of ferric oxide nano/micro particles has been carried out by using solvothermal route. Structural characterization displays that the predominant morphologies are porous hollow spheres, microspheres, micro rectangular platelets, octahedral and irregular shaped particles. It is also observed that solvent has significant effect on morphology such as shape and size of the particles. All the morphologies obtained by using different solvents are nearly uniform with narrow size distribution range. The values of full width at half maxima (FWHM) of all the products were calculated to compare their size distribution. The FWHM value varies with size of the particles for example small size particles show polydispersity whereas large size particles have shown monodispersity. The size of particles increases with decrease in polarity of the solvent whereas their shape changes from spherical to rectangular/irregular with decrease in polarity of the solvent. The catalytic activities of all the products were investigated for both dry and wet processes such as thermal decomposition of ammonium per chlorate (AP) and reduction of 4-nitrophenol in aqueous media. The results indicate that each product has a tendency to act as a catalyst. The porous hollow spheres decrease the thermal decomposition temperature of AP by 140 °C and octahedral Fe_3_O_4_ particles decrease the decomposition temperature by 30 °C. The value of apparent rate constant (k_app_) of reduction of 4-NP has also been calculated.

## Background

Magnetic nano materials possess unique prospects in various fields of life due to their well-regulated size and magnetic properties [1]. Iron oxide magnetic nano spheres are inclined to be either paramagnetic or super paramagnetic with a size fluctuating from a few nanometers to tens of nanometers. Iron oxide nanoparticles are of pronounced curiosity for investigators from a wide range of disciplines like magnetic fluids [2], catalysis [3], biotechnology/biomedicine [4], magnetic resonance imaging [5], data storage [6] and environmental remediation [7]. Functionalized nanoparticles are very encouraging for applications in catalysis [8], bio labeling [9], and bio separation [10]. Specifically in liquid-phase catalytic reactions, such small and magnetically separable particles are very useful because quasi homogeneous systems possess advantage of high dispersion, high reactivity and easy separation [11, 12]. These magnetic nanoparticles possess high magnetic moment which helps to efficiently bind the specific biomolecules under physiological conditions. These nanoparticles often display very stimulating electrical, optical, magnetic and chemical properties, which cannot be attained by their bulk complements.

It is well-known that the properties of nano materials are strongly dependent on their morphology and structure. That’s why different morphologies including nanorods, [13, 14] nanotubes [15] and nanospheres [16, 17] of ferric oxide nano materials have gained considerable attention. As one of the most important, non-toxic, nature-friendly, corrosion-resistant and stable metal oxide, hematite (Fe_2_O_3_) has become a very attractive material due to its wide applications in various fields [18]. Hydrothermal [19], microwave hydrothermal [20] and microwave solvothermal [21] methods are truly low temperature methods for the preparation of nanoscale materials of different size and shape. These methods save energy and are environmentally benign because these reactions take place in closed system conditions. Synthesis of monodisperse nanometer-sized magnetic particles of metal alloys and metal oxides are an active research area because of their potential technological ramifications ranging from ultrahigh-density magnetic storage media to biological imaging. Size, size distribution, shape, and dimensionality are important for the properties of these magnetic materials [22, 23]. Nanoparticles of various iron oxides (Fe_3_O_4_ and ç-Fe_2_O_3_ in particular) have been widely used in a range of applications. Iron oxide nanoparticles have been used as catalyst for thermal degradation of ammonium perchlorate (AP) and reduction of nitrophenols. Campos et al. studied the thermal degradation of AP in the presence of Fe_2_O_3_ catalyst [24]. Xu et al. used Fe_2_O_3_ microoctahedrons and nanorods as catalyst for thermal degradation of AP [25]. Alizadeh-Gheshlaghi et al. compared the catalytic activity of copper oxide, copper chromite and cobalt oxide nanoparticles [26]. They found that copper chromite shows best catalytic activity among all samples because these nanoparticles decrease the thermal decomposition temperature of AP by 103 °C. Scientists have reported effect of size of nanoparticle on catalysis. But they did not report the effect of nature and composition of solvent on size and morphology of ferric oxide (Fe_3_O_4_) particles and their catalytic properties. This is the novelty of this work. Here we are introducing template free synthesis of magnetite (Fe_3_O_4_) micro and nanoparticles at low temperature and effect of morphology and size of particles on their catalytic properties.

In this article, nano/micro particles of different morphology are prepared by using different solvents and mixture of solvents to carry out a comparative study. Synthesized products are characterized by XRD, SEM and TEM. A diverse range of products are obtained like sphere, spherical aggregate, irregular, micro rectangular platelet and octahedron. The catalytic activity of all particles is also studied in dry as well as in wet media. The effect of morphology and size of Fe_3_O_4_ particles on catalytic activity is investigated and compared with each other.

## Experimental

### Materials

All the chemicals are purchased commercially and used without any further purification. Ferric chloride (FeCl_3_•6H_2_O), sodium borohydride (NaBH_4_), sodium ethanoate, poly ethylene glycol, n-hexane, absolute alcohol, ammonium perchlorate, 4-nitrophenol (4-NP), and ethylene glycol (EG) are utilized for the synthesis of nano/micro particles. Deionized water is used throughout the experimental work.

### Synthesis of different morphologies of ferric oxide nano/micro particles

1.35 g of FeCl_3_•6H_2_O was dissolved in 30 mL of ethylene glycol and 3.6 g of sodium ethanoate was dissolved in 30 mL of ethylene glycol separately. Then both solutions were stirred for 10 min separately. Later both solutions were mixed with each other and allowed to stir for 30 min. After 30 min, a black liquid was transferred to Teflon lined autoclave of 100 mL capacity. The autoclave was sealed at a constant temperature of 200 °C for 18 h. After heating, the autoclave is allowed to cool at room temperature. Product was collected by centrifugation at 3000 rpm. The resulting product was washed three times with deionized water and three times with absolute alcohol. The washed precipitates were dried in a vacuum oven at 60 °C for 12 h. In this way product A was obtained. Similarly product B is synthesized by using the same protocol as mentioned above but the solvent ethylene glycol was replaced by deionized water and ethylene glycol (1:1) ratio. The product C is prepared by using polyethylene glycol as solvent whereas n-hexane is used as solvent for the synthesis of product D. The product E was synthesized by using a mixture of n-hexane and ethylene glycol (1:1) as solvent. The details of solvents and their appropriate ratios are given in Table 1.

**Table 1:** Comparison of effect of nature and composition of solvent on morphology and size of Fe_3_O_4_ particles and their catalytic properties

Product	Solvent (s)		Nano/micro structure (s)		Catalytic thermal decomposition of AP			k_app_ of catalytic reduction of 4-NP
	Composition	Ratio	Morphology	Size	Final decomposition temperature (°C)	Temperature of maximum loss in mass percentage (°C)	Decrease in final decomposition temperature (°C)	
A	Ethylene glycol	100%	Porous hollow sphere	140 nm	310	285	140	0.4206**/**min
B	Deionised water: ethylene glycol	1:1	Microsphere	415 nm	345	329	105	0.3073/min
C	Poly ethylene glycol	100%	Micro rectangular platelet	~12 µm	390	373	60	0.3054/min
D	n-Hexane	100%	Octahedron	~4.3 µm	420	387	30	0.2834/min
E	n-Hexane: ethylene glycol	1:1	Irregular	~4 µm	400	360	50	0.2837/min

### Catalytic activity

Catalytic activity in thermal decomposition of AP is studied for all the prepared samples by adding only 1% catalyst in AP. A mixture of catalyst A and AP was prepared by mixing 0.1 g of catalyst and 9.9 g of AP. Mixture of catalyst and AP was ground to ensure the proper mixing. Further thermal decomposition was monitored with NEZSCH TGA.

1.8 mL of 0.111 mM 4-NP, 0.5 mL of 50 mM NaBH_4_ and catalyst were added in a cuvette and spectrum was scanned in 200–500 nm wavelength range. The spectra were scanned on UVD3500 spectrophotometer. The spectra were scanned after every minute till absorbance at 400 and 300 nm becomes constant.

### Structural characterization

X-ray powder diffraction (XRD) patterns were obtained on a Rigaku D/max Ultima III X-ray diffractometer with a Cu-Kα radiation source (λ = 0.15406 nm) operated at 40 kV and 150 mA at a scanning step of 0.02° in the 2θ range 10–80°. Scanning electron microscopy observation was performed on a JEOL JSM-6480A scanning electron microscope. Transmission electron microscopy (TEM) observation was performed on an FEI Tecnai G2 S-Twin TEM with an accelerating voltage of 200 kV. Thermo gravimetric was taken on NEZSCH STA 409 PC with a heating rate of 10 °C/min from 50 to 600 °C. UVD3500, Shimadzu was used to monitor the catalytic reduction of 4-NP.

## Results and discussion

### Structural characterization

#### XRD analysis

**Fig. 1:**
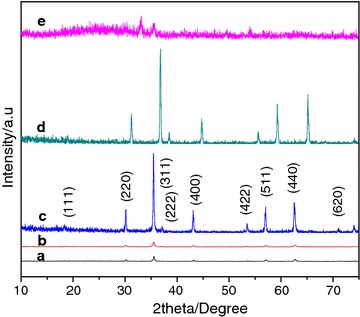
XRD patterns of as-prepared Fe_3_O_4_. XRD patterns *a, b, c, d* and *e* correspond to product A–E respectively

XRD patterns of all synthesized products are shown in Fig. 1. XRD data analysis shows that product is Fe_3_O_4_. The position and relative intensity of all diffraction lines match well with those of the commercial magnetite powder (Aldrich catalog No. 31,006-9) reported by Sun et al. [27]. Various parameters are obtained through XRD data analysis whose detail is given in Table 2. Space group, unit cell type, coordination number, position of atoms, cell parameters, d-spacing and miller indices (hkl) values are summarized in this table. Diffraction lines analysis of Fig. 1a and b indicates that product A and B possess monoclinic unit cell structure. Diffraction lines analysis of Fig. 1c and d indicates that product C and D possess face centered cubic unit cell structure. Lin et al. and Mckenna et al. had also analyzed that Fe_3_O_4_ is made up of cubic unit cells [28, 29]. Wright et al. had analyzed that Fe_3_O_4_ is made up of monoclinic unit cells [30]. Absence of any extra peak in the XRD patterns shows that obtained product obtained is highly pure. Sharp and strong diffraction lines confirmed that product is highly crystalline.

**Table 2:** Summary of various parameters obtained from XRD pattern analysis of products A–E

Parameter	Product C and D	Product A and B
Name of compound	Magnetite	Magnetite
JCPDS no.	19-0629	28-0491
Crystal system	Cubic	Monoclinic
Type	Face centered	Primitive
Space group	Fd-3 m (227)	P12/m1 (10)
Crystallite size (Å)	282	282
Cell parameters		
a, b and c (Å)	8.3851, 8.3851 and 8.3851	5.9444, 5.9247 and 8.3875
α, β and γ (°)	90.0, 90.0 and 90.0	90.0, 90.237° and 90.0
Atom coordinates		
x, y and z of iron	0.125, 0.125 and 0.125	0.750, 0.500 and 0.125
	0.500, 0.500 and 0.500	0.000, 0.500 and 0.000
		0.250, 0.250 and 0.250
		0.000, 0.000 and 0.500
		0.500, 0.500 and 0.000
		0.500, 0.000 and 0.500
		0.750, 0.000 and 0.125
x, y and z of oxygen	0.253, 0.253 and 0.253	0.250, 0.260 and 0.005
		0.510, 0.500 and 0.755
		0.250, 0.240 and 0.495
		0.010, 0.000 and 0.255
		0.510, 0.000 and 0.745
		0.010, 0.500 and 0.245
No. of formula units per unit cells (Z)	8.0	4.0
Density (g/cm^3^)	5.21600	5.2060
Volume (Å^3^)	591.9	225.6
Spacing (d_hkl_) (Å), 2-theta (°) and miller indices (hkl)	4.84743, 18.286 and (111)	5.43, 16.310 and (010)
	2.96843, 30.079 and (220)	4.05653, 21.892 and (100)
	2.53149, 35.429 and (311)	2.88045, 31.021 and (101)
	2.42372, 37.061 and (222)	2.715, 32.963 and (020)
	2.09900, 43.058 and (400)	2.69153, 33.259 and (002)
	1.9261, 47.144 and (331)	2.59659, 34.513 and ($\bar{1}0 2$ )
	1.71383, 53.416 and (422)	2.20488, 40.895 and ($\bar{1}21$)
	1.61581, 56.942 and (333)	1.78442, 51.147 and ($\bar{2}12$)
	1.48422, 62.527 and (440)	1.74586, 52.361 and (201)
	1.41918, 65.743 and (531)	1.65292, 55.551 and (130)
	1.39933, 66.797 and (442)	1.63239, 56.311 and ($\bar{1}31$)
	1.32752, 70.934 and (620)	1.39209, 67.190 and (212)
	1.28038, 73.969 and (533)	1.3575, 69.141 and (040)
	1.26574, 74.970 and (622)	1.34287, 70.004 and (132)
		1.30996, 72.033 and (123)
		1.28733, 73.504 and (140)
		1.27756, 74.160 and ($\bar{1}41$)
		1.24264, 76.613 and ($\bar{1}24$)
		1.23355, 77.282 and (301)
		1.21037, 79.047 and (320)

#### SEM and TEM observations

**Fig. 2:**
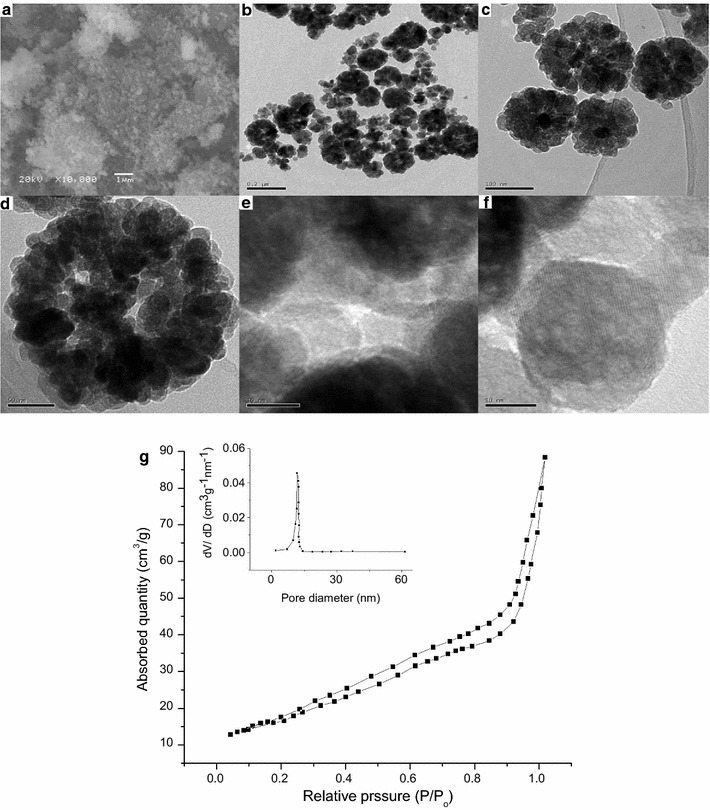
**a** SEM images of Fe_3_O_4_ prepared, **b** TEM image of product, **c** hollow spherical aggregates, **d** spherical aggregate, **e** and **f** HRTEM images of the product. **g** Nitrogen adsorption–desorption isotherm and corresponding BJH pore-size distribution curve of product A

The morphology and structure of obtained products were investigated by SEM and TEM as shown in Fig. 2 for five different products prepared. The comparison of products obtained on the basis of solvent used in solvothermal process is given in Table 1.

#### Product A: porous hollow spheres of Fe_3_O_4_

SEM and TEM images of product A are given in Fig. 2. Figure 2a shows an overview of the product. It seems from this image that size of particles is very small and formed aggregates. Therefore it is difficult to differentiate the morphology of the product and estimate the average size of particles by SEM. Thus TEM was carried out to investigate the exact morphology. TEM micrographs (Fig. 2b–d) show that the product is nearly spherical in shape. It is also observed that very small nanoparticles (~10 nm) have assembled together and formed a spherical morphology. But these spheres are not very uniform. These aggregates of nanoparticles appear to be hollow from inside. Figure 2b also confirms the presence of hollow spheres with a wide opening at the apical surface (indicated by red arrow in the Fig. 2b). The product Fe_3_O_4_ is formed by loose packing of nanoparticles, thus small pores have left behind (Fig. 2d). The average size of these hollow spheres is approximately 140 nm. Few spheres are also present in product whose size is smaller or bigger than 140 nm. Some of the spherical aggregates might have broken because small nanoparticles are visible in microscopic images.

HRTEM images of the Fe_3_O_4_ microspheres and nano spheres obtained is shown in Fig. 2e and f. It can be seen that the nanoparticles organized so well that they assembled into a single crystal by sharing identical lattices, though some open pores and defects in HRTEM images of the Fe_3_O_4_ microspheres are also observed. These are obvious boundaries of the assembled small Fe_3_O_4_ nanoparticles. The particles of product A are hollow from inside confirmed by SEM and TEM observations. This result shows that the spherical morphology obtained when ethylene glycol was used as solvent and the size of product obtained is uniform. The hollow sphere and porous structure might be result of carbon dioxide or methane gas trapped inside these spheres. With the increase in heating time the gas pressure inside the spheres increased that increased the size of spheres and finally this gas comes out leaving behind an opening and pores on the surface of these hollow porous spheres. The porosity of these structures is also analyzed by nitrogen adsorption–desorption isotherm. This isotherm is given as Fig. 2g. This plot indicates that product is porous. The specific surface area of this product is calculated as 35.63 m^2^/g.

#### Product B: microspheres of Fe_3_O_4_

**Fig. 3:**
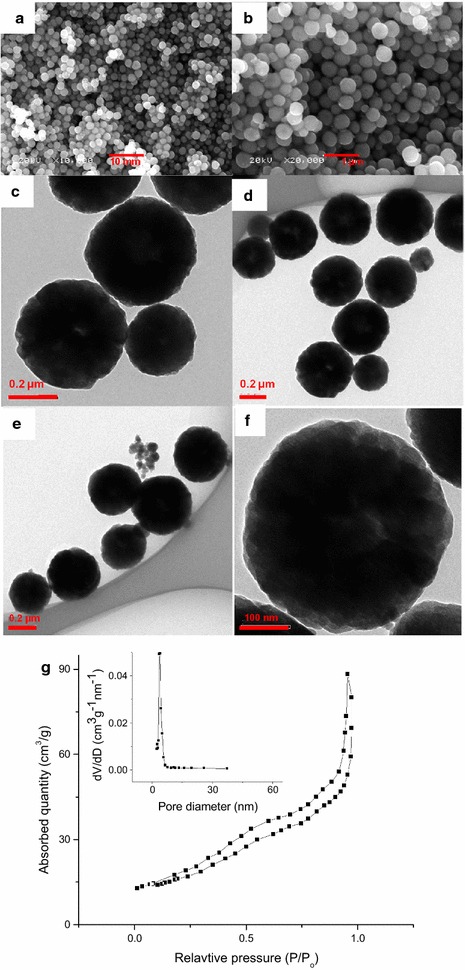
SEM and TEM images of product B, **a**–**c** SEM overview of the microspheres, **d**, **e** TEM overview of microspheres, and **f** a single microsphere. **g** Nitrogen adsorption–desorption isotherm with the corresponding BJH pore-size distribution curve (the *inset*) of product B

The product B is obtained by using deionized water and ethylene glycol, in a ratio of 1:1, as solvent. The product B is characterized by using SEM and TEM and the results are shown in Fig. 3. The SEM observation shows that product is fairly spherical with no opening. The size of these particles is in range of 140–415 nm but most of them are about 415 nm. The product is appeared as bulk and clustered together due to very large amount of spherical particles present among the product B as shown in Fig. 3a–c.

TEM observations, shown in Fig. 3d–f, are in good agreement with the results obtained by SEM images. The product B is uniformly spherical with distinct boundaries and compact shape. No irregularities have observed in the morphology of the product. The average size of the product measured by TEM micrograph is approximately 415 nm whereas a few nanospheres are also appeared along with these microparticles.

The edges of these microparticles are very sharp with no zigzag which confirms that the product B is uniformly spherical in shape. The TEM images show the contrast of light and dark colors that either confined to the presence of very thin walls/boundaries of the microspheres or indicating the presence of cavity inside the spheres. These spheres might be hollow from inside but no broken microsphere has observed in SEM and TEM micrographs to confirm the presence of hollow microspheres. Nitrogen adsorption–desorption isotherm is used for analysis of porosity of product B (Fig. 3g). This plot shows that product is porous. BET pore size distribution is also calculated as 22.9 m^2^/g.

#### Product C: micro rectangular platelets of Fe_3_O_4_

**Fig. 4:**
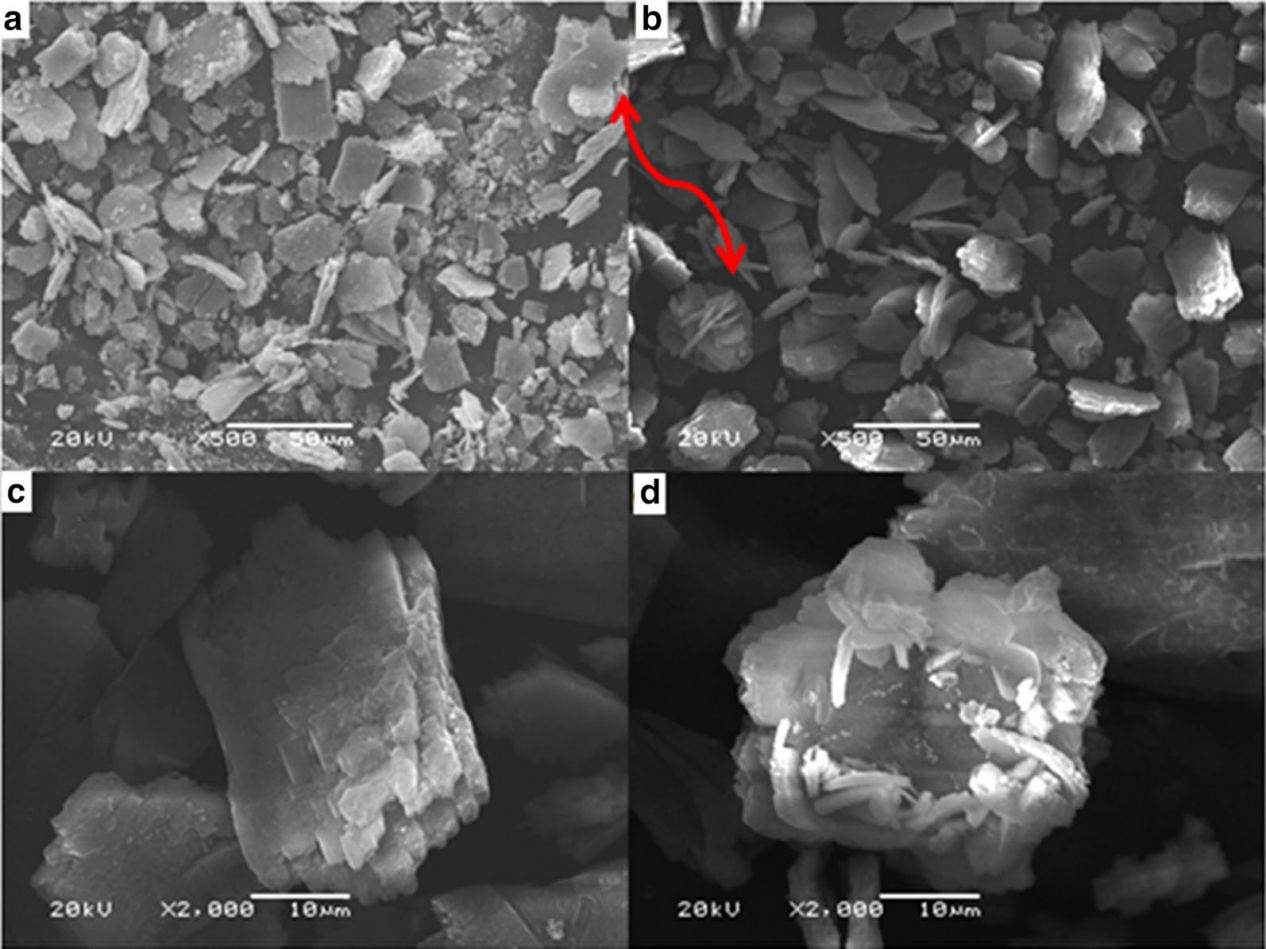
SEM observations of micro rectangular platelets (product C) of Fe_3_O_4_, **a** and **b** an overview of the product, **c** micro rectangular platelets of Fe_3_O_4_, **d** flower like structure formed by discs

The product obtained by using poly ethylene glycol as solvent in solvothermal method named as product C. It has characterized by SEM and TEM and obtained results are shown as Fig. 4. It is evident from Fig. 4a and b that the product is consisted of micro rectangular platelets (flakes). It seems that particles align together in layer-by-layer assembly and form these platelets. The size of these one dimensional rectangular platelets or petals is ranging from 10 to 20 µm in length and 8–12 µm in width. These platelets are multi layered think that is approximately 5 µm as shown in Fig. 4c. These rectangular platelets show a specific trend of assembling, as indicated by red arrow in Fig. 4a and b. This assembly of the platelets is slightly appeared like some flower shaped morphology in which these platelets act as petals. These platelets are interlinked from the middle and give a shape as that of cross as shown in Fig. 4a (at one end of two sided red arrow). This cross followed by the addition of further platelets and acquires a shape of flower as shown in Fig. 4b (another end of red arrow). This layer by layer arrangement of these platelets finally leads to a flower like morphology that appeared in Fig. 4d. The edges of this flower shape Fe_3_O_4_ are very similar to that of original flowers and some of the platelets oriented upwards acts as stamens (middle portion of original flowers). There are two possibilities about this product C: (1) firstly flower like structures are formed but by heating further these structures are broken and give rise to the rectangular layer by layer assembled platelets: (2) the rectangular platelets are formed and arrange in a specific pattern to give rise to flower like structure. At the current conditions of experiment, the main product is micro rectangular platelet.

#### Product D: octahedra of Fe_3_O_4_

**Fig. 5:**
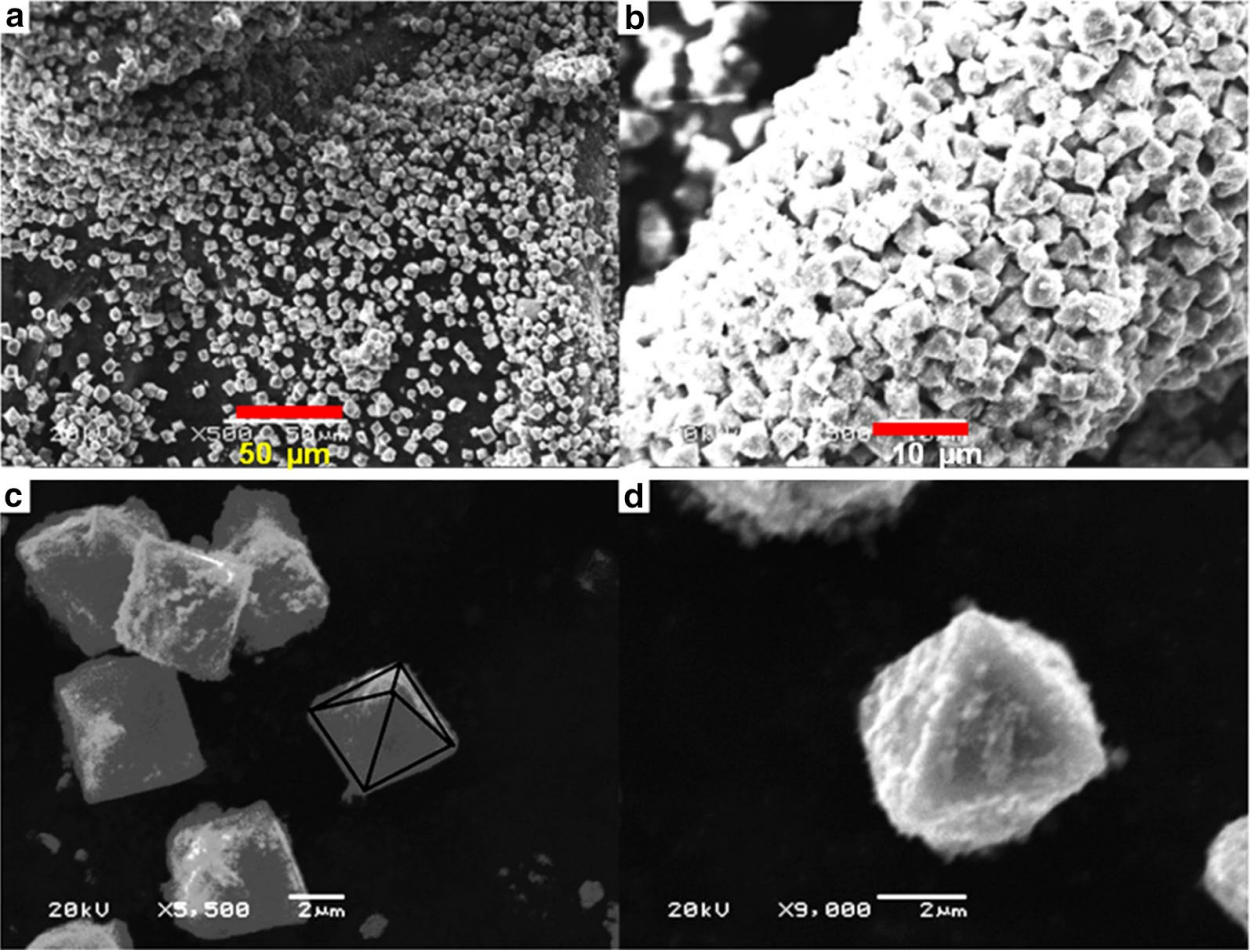
SEM observations of octahedral microparticles (product D), **a** an overview of the product, **b** octahedral particles aggregated together in the form of cylindrical rod, **c** different octahedral particles, **d** single octahedral structure

The product D was obtained by using n-hexane as solvent. It morphology was characterized by SEM. The results are shown in Fig. 5a–d clearly indicate the presence of polyhedron morphology. The product consists of uniform sized octahedral microparticles with eight distinct faces. These particles are not present in the form of aggregates but separated from each other as shown in Fig. 5a but b shows the aggregate of these octahedral particles. These octahedral particles are aligned together in the form of long cylinder. The size of these octahedrons is uniform throughout the product with no variations.

The size of each face of this octahedron is approximately 2.5 µm and the average diameter from one end to another is almost 4.3 µm. A few nanometer sized particles attached on the surface of these micro octahedra are observed in SEM micrograph Fig. 5. These micro octahedra appear to be very compact and rigid from outer surface as well as from inner surface. The edges of these octahedron are uniform and distinct with no irregularities are observed.

It might be some cubic shaped particles that appeared first that further grows towards the edges (each face of polyhedron). The lattice cell appeared at the initial of the reaction and solvent molecule surrounds it in a specific pattern that facilitates its growth to an octahedral micro particles. It is concluded from the fact, n-hexane is utilized as solvent in solvothermal synthesis support the octahedral morphology.

#### Product E: irregular morphology of Fe_3_O_4_

**Fig. 6:**
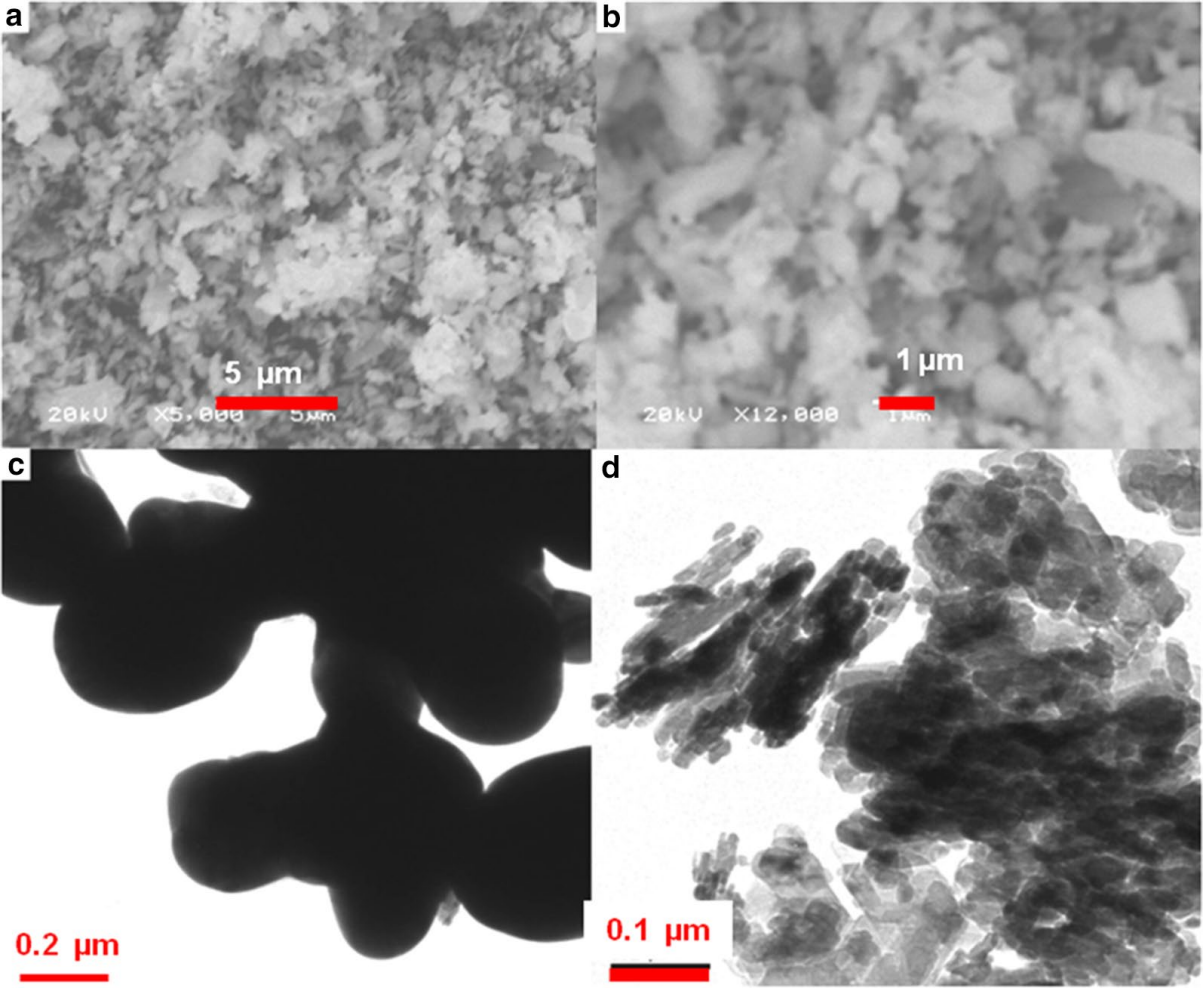
SEM and TEM observations of irregular shaped Fe_3_O_4_ particles, **a** and **b** SEM images of the product E, **c** and **d** TEM images of the product

To prepare the product E, n-hexane and ethylene glycol in a ratio of 1:1 was used as solvent under solvothermal conditions. The product obtained is further dealt with structure characterization by using SEM and TEM and the results are given as Fig. 6a–d. Product E shows irregular geometry when it is examined through the SEM. Some of the particles are irregular shaped embedded in some material. Under the low resolution of SEM, it is not possible to differentiate between different shapes appeared in the product rather than any uniform shape and morphology. For a clear indication of the structure of Fe_3_O_4_ particles, TEM is carried out. The results are given as Fig. 6c and d. Some irregular shaped particles are of few micrometers size and some of them are connected like net and run to several micro meters. Besides these big particles, there are present a large number small particles.

#### Effect of nature and composition of solvent on size and size distribution of products

**Fig. 7:**
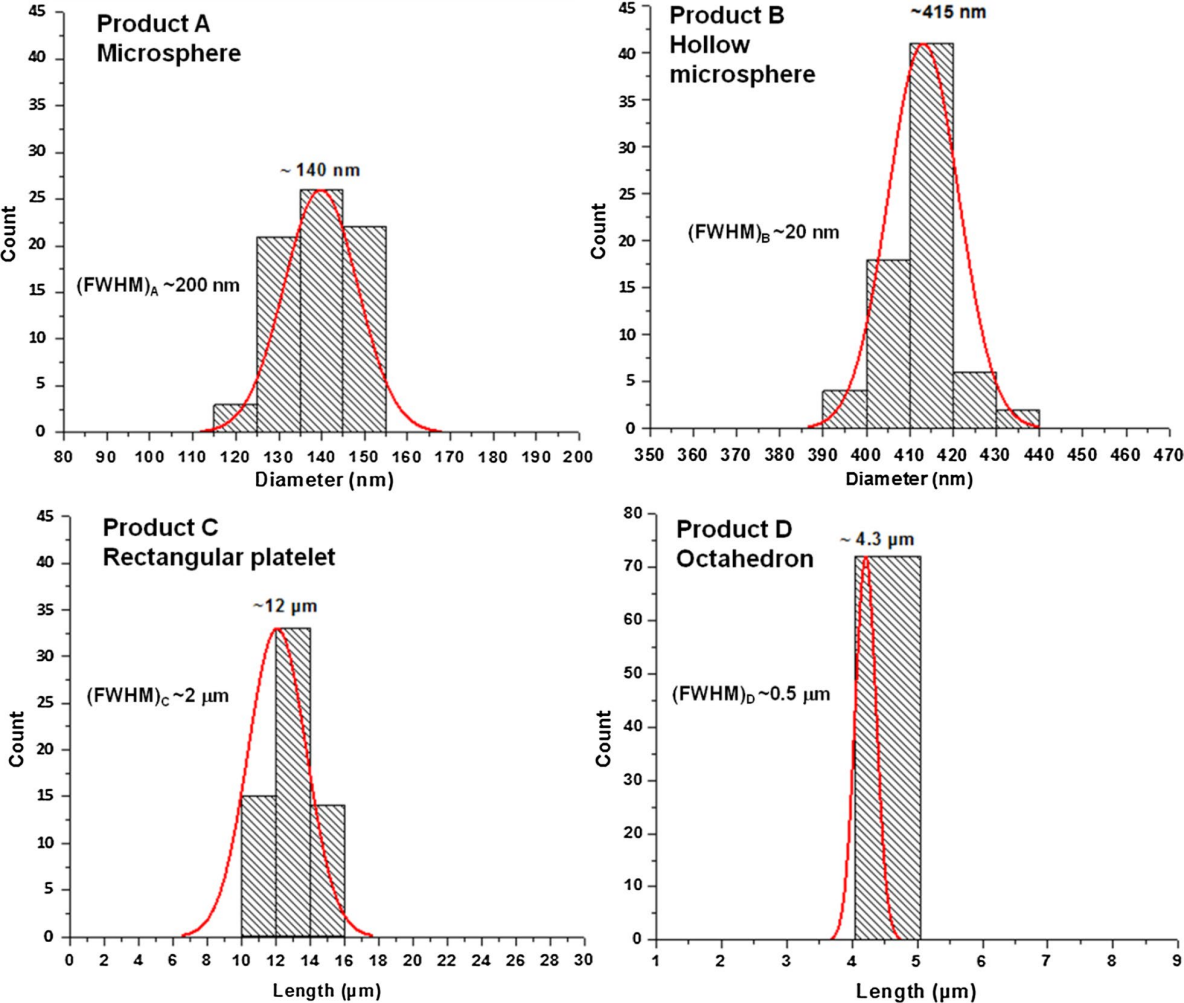
Size distribution histograms of synthesized product A–D

The size distribution histograms of products A–D are given in Fig. 7. This figure shows that the particle size of products is in order: A<B<C<D<E. Non-polar solvent n-hexane was used for synthesis of product E while less non-polar solvent ethylene glycol was used for synthesis of product A. Polarity of solvent used during synthesis is decreases from product A to D. It means particles of smaller size are synthesized using less non-polar solvent and particles of larger size are synthesized using more non-polar solvent. The size distribution of products A–D can be compared from Fig. 7. Size distribution histogram of product E is not given because product E possess irregular reef like structures (as confirmed from SEM images of Fig. 6). All the size distribution histograms obeyed Gaussian distribution and possess one peak only. It means the size of particles of products A–D vary in a specific range only. Gaussian distribution shows that particles of products A–D possess homogenous size distribution. It means that products A–D are monodisperse. The full width at half maxima (FWHM) value of all products was also calculated and given in Fig. 7. FWHM value of product A and B can be compared with each because both products contain particles above 100 nm. Similarly FWHM value of product C and D can be compared with each other because both products contain particles below 100 μm. (FWHM)_B_ is smaller than (FWHM)_A_ which shows that product B possess narrower size distribution than that of product A. This is due to the lesser polarity of solvent of product A than that of product B. Mixture of two solvents (ethylene glycol and water) was used for synthesis of product B while pure ethylene glycol was used for synthesis of product A. Microparticles of product B was synthesized on organic-water interface, that’s why product B possess narrower distribution than that of product A. On the other hand, value of (FWHM)_D_ is smaller that of (FWHM)_C_ because polarity of solvent used for synthesis of product D is lesser than that of product C. The size distribution of graphs is compared from their respective value of FWHM. It means size of particles decreases with increase in polarity while FWHM value increases with increase in polarity. If smaller size is obtained then size distribution becomes large and if narrow size distribution is achieved then size of particles become greater. Hence compromise on size or distribution of particles is to be made.

### Catalytic activity

The catalytic activity of Fe_3_O_4_ nano/micro particles was investigated for dry as well as wet media processes. Fe_3_O_4_ nano/micro particles was used to catalyze the thermal degradation of AP as dry media process and reduction of 4-NP as wet media process.

#### Catalytic thermal of degradation of ammonium perchlorate

**Fig. 8:**
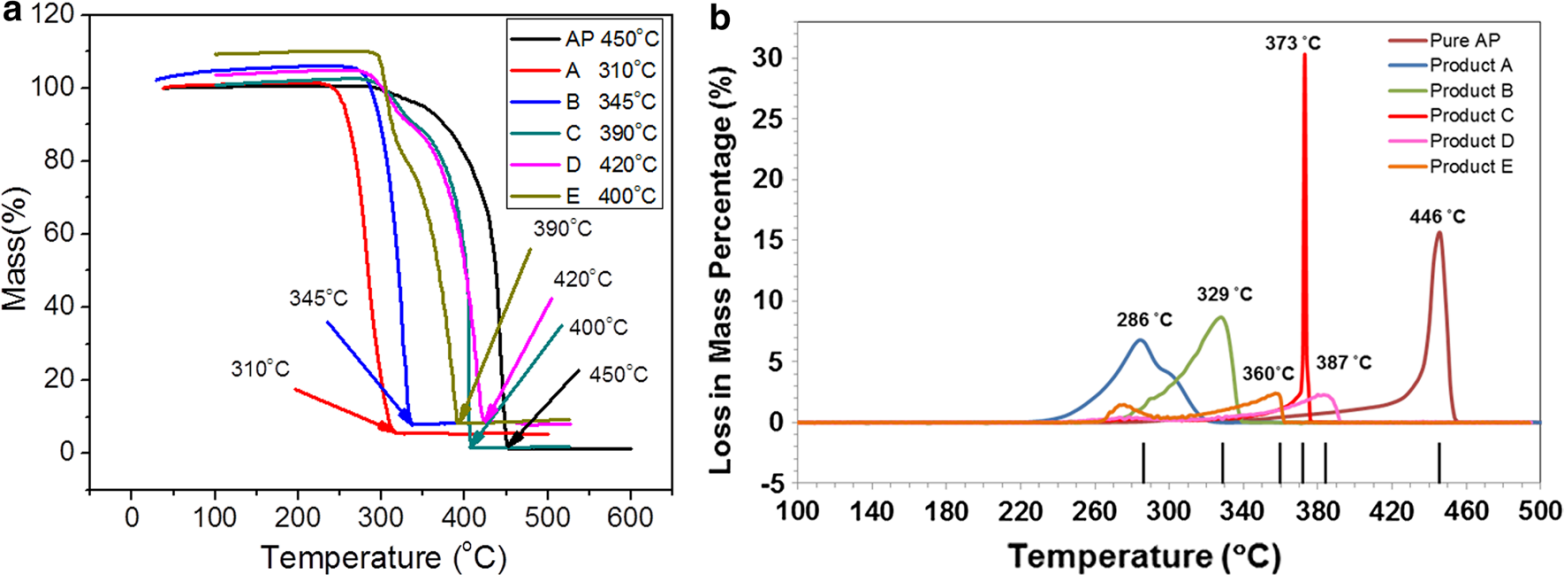
**a** TG observations of decomposition of AP in the presence of Fe_3_O_4_ particles of different morphologies, and **b** temperature dependent plot of loss in mass percentage of AP in the presence of Fe_3_O_4_ particles of different morphologies

The catalytic thermal decomposition of AP is carried out by using the thermal gravimetric analysis (TG) (Fig. 8a). Thermal decomposition temperature of pure AP is 450 °C. It is observed that all the synthesized catalysts have shown considerable catalytic activity. The thermal degradation of AP is based on proton transfer mechanism. The degradation of the AP starts with the transfer of charge among reactants. This charge transfer process is a high energy phenomenon. The thermal energy provides energy to the charges to overcome the barrier and transform the reactants into products. The Fe_3_O_4_ nano/micro particles facilitate this charge transfer process. So charges cross the barrier at low temperature in the presence of catalyst and convert the reactants into products. The same mechanism is also proposed by Chaturvedi et al. and Dey et al. for thermal degradation of AP in the presence of metals [31, 32].

The catalyst A, porous hollow spheres with almost 140 nm diameter are proved to be the best among all of these catalysts. It is shown in graph that final decomposition temperature for the porous hollow spheres is 310 °C. There is almost 140 °C decrease in thermal decomposition temperature of AP when porous hollow are used as catalyst. The thermal decomposition curve for this process is very smooth without any irregularities. Octahedral particles (catalyst D) showed lowest catalytic activity among all catalysts. The final decomposition temperature of AP is measured to 420 °C in the presence of this catalyst. There is a decrease of 30 °C in the final thermal decomposition of AP. The other catalysts with their thermal decomposition temperatures are given in Table 1.

Loss in mass percentage of AP versus temperature is shown in Fig. 8b. The extent of decomposition of AP is clearly shown in this figure. This figure shows that the temperature, at which maximum loss in mass percentage AP has occurred, is different for different catalysts. Catalyst C (micro rectangular platelets) catalyzed decomposition is most significant because all the mass of AP decomposed at once when temperature reached 373 °C. While in case of remaining all the catalysts, decomposition of AP is not at once. After catalyst C, catalysts A (hollow microspheres) and B (microspheres) also shows a sharp loss in mass percentage of AP at temperature 329 and 286 °C respectively. But catalysts D and E show no peak in Fig. 8b, it means a continuous decrease in mass of AP occur over whole temperature range of decomposition.

Catalyst A shows maximum decrease in thermal decomposition temperature of AP among all the catalysts. While catalyst C shows sharp loss in mass percentage of AP at temperature 373 °C among all the catalysts. Size of particles of catalyst A is smallest among all catalysts and it shows good catalytic activity. Hence product A can be considered as a best catalyst among all the synthesized catalysts.

#### Catalytic reduction of 4-nitrophenol

**Fig. 9:**
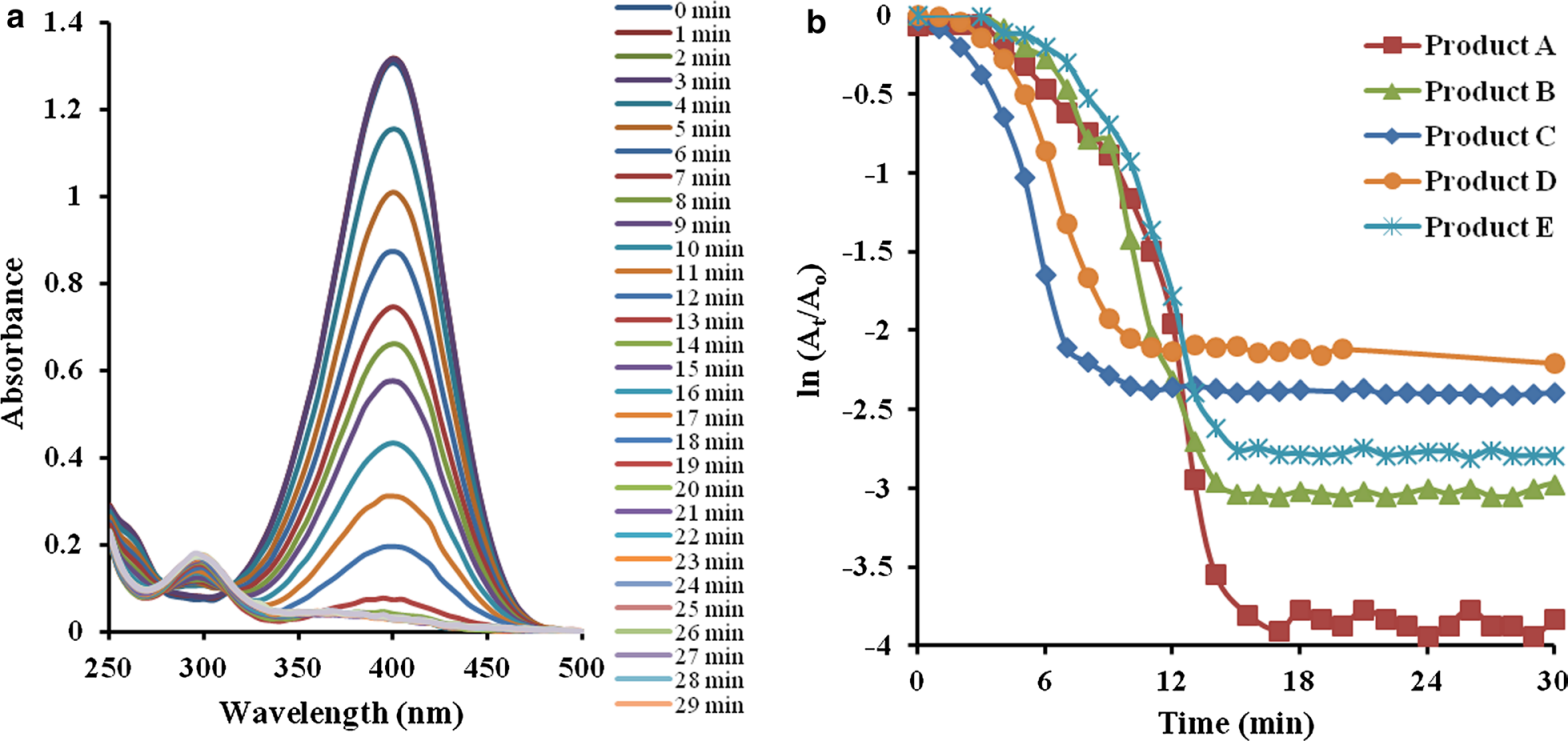
**a** Time dependent UV–Visible spectra of reduction of 4-NP catalyzed by product A in aqueous medium. **b** Plot of ln(A_t_/A_0_) versus time for reduction of 4-NP catalyzed by product A–E [conditions: [4-NP] = 80 μM, [NaBH_4_] = 8 mM, [Fe_3_O_4_] = 1 μg/L and temperature = 22 °C]

Reduction of 4-NP in aqueous media is used as a model process to investigated the catalytic activity of Fe_3_O_4_ micro/nano particles in wet media. Fe_3_O_4_ nano/micro particles catalyzed the reduction of 4-NP into 4-aminophenol (4-AP). 4-NP and 4-AP both absorb in UV–Visible region because λ_max_ of 4-NP and 4-AP are 400 and 300 nm respectively [33]. That is why the reduction of 4-NP is monitored by UV–Visible spectrophotometery. Catalytic reduction of 4-NP in the presence of excess of reducing agent NaBH_4_ obeys pseudo first order kinetics. Its kinetic equation is ln(A_t_/A_0_) = –k_app_ × t (where A_0_ and A_t_ are absorbance of 4-NP at time 0 and t and k_app_ is apparent rate constant of reduction). Time dependent UV–Visible spectra of reduction of 4-NP catalyzed by catalyst A (hollow microsphere) is shown in Fig. 9a. It is clearly visible from this figure that only one specie is present in reaction mixture at time 0 min because spectra possess only one peak at 400 nm. This shows that 4-NP was present in reaction mixture initially. As soon as the reduction of 4-NP progresses, the absorbance at 400 nm is started to decrease while absorbance at 300 nm is started to increase.

**Fig. 10:**
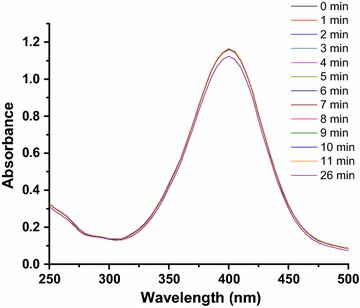
**a** Time dependent UV–Visible spectra of reduction of 4-NP in the absence of catalyst [conditions: [4-NP] = 80 μM, [NaBH_4_] = 8 mM and temperature = 22 °C]

The catalytic reduction of 4-NP is also studied in the absence of catalyst (Fig. 10). It is observed that absorbance at 400 nm did not change appreciably till 26 min. This shows that Fe_3_O_4_ catalyst facilitates the reduction of 4-NP, that is why the absorbance at 400 nm is decreased to 0.6 after 26 min in the presence of catalyst (Fig. 9a). Plot of ln(A_t_/A_0_) as a function of time of reduction of 4-NP catalyzed by catalysts A–E is shown in Fig. 9b. The reduction of 4-NP catalyzed by all catalysts A–E was studied under same catalyst dosage, reactants concentration and temperature, so that the effect of particle morphology on apparent rate constant (k_app_) can be easily investigated. Initially the value of ln(A_t_/A_0_) does not decrease with time in all plots. This duration is known as induction period. Then value of ln(A_t_/A_0_) is started to decrease with time which shows that catalytic reduction is in progress. Later the value of ln(A_t_/A_0_) becomes constant with the passage of time which shows that reaction has completed. The linear region of the plot of ln(A_t_/A_0_) versus time was used to calculate the value of k_app_ of reduction. The calculated values of k_app_ for the reactions catalyzed by catalyst A–E are given in Table 1. These values of k_app_ are in the following order: A>B>C>D>E. This might be due to the difference in their size and morphology. The size of product decreases in the following order: A<B<C<D<E. It is well known that catalysis is a surface phenomenon. The surface area of particles decreases with increase size. So number of active sites decrease with increase in size. If small number of active sites are present then small number of reactant molecules will adsorb and value of k_app_ decrease resultantly. The value of k_app_ of reduction catalyzed by catalyst A (porous hollow spheres) is greatest among all the products. Product A is porous and possesses very small size, so it provides very large surface area for catalysis. That is why it shows maximum value of k_app_ than that of all. The value of k_app_ of catalysts D and E is almost same because their sizes are almost same. This also confirms that value of k_app_ depends upon size.

## Conclusions

The predominant morphologies of the Fe_3_O_4_ particles synthesized are hollow nanospheres and microspheres. Although other shapes including spherical aggregates, octahedra, irregular structures and micro rectangular platelets are also prepared by using different solvents including ethylene glycol, water, n-hexane in different ratios. Most of the products of Fe_3_O_4_ prepared are uniform in shape and size distribution, well separated from each other and hollow from inside with thin but definite boundaries. The catalytic activity of all the synthesized catalysts is investigated for thermal decomposition of AP. The results show that catalysts have very good surface properties. Fe_3_O_4_ catalysts show a trend in catalytic thermal decomposition of AP. With increase in size of Fe_3_O_4_ particles, the catalytic properties gradually decrease and particles with 140 nm size decrease the decomposition temperature by 140 °C. It was also investigated that the temperature at which maximum loss in mass percentage of AP occurred. All the AP decomposed at once at 373 °C by micro rectangular platelets catalyst. The rest of all catalysts catalyzed the continuous decomposition of AP over the complete range of temperature. All the catalysts are also used as catalyst for reduction of 4-nitrophenol. It is observed that value of k_app_ of reduction is highest for catalyst hollow microspheres and lowest for catalyst rectangular platelets. It is also observed that value of k_app_ is decreased with increase in size of particles. The above results have shown that these catalysts can be efficiently used for dry as well as wet processes.
